# Family Health Conversations have Positive Outcomes on Families - A Mixed Method Research Study

**DOI:** 10.2174/1874434601711010014

**Published:** 2017-02-28

**Authors:** Åsa Dorell, Ulf Isaksson, Ulrika Östlund, Karin Sundin

**Affiliations:** 1 Umea University, Department of Nursing, Campus Ornskoldsvik, Box 843, SE-901 87 Umeå, Sweden; 2 Centre for Research & Development, Uppsala University/Region Gävleborg, Gävle, Sweden

**Keywords:** Family Hardiness Index, Family Health Conversation, Family Systems Nursing, Intervention, Mixed methods research design, Older people, Transition, Quality of Life

## Abstract

**Background:**

Having a family member living in a residential home affects the entire family and can be hard to handle. Family members require encouraging and open communication support from nurses during and after relocation to a residential home. A Family Systems Nursing intervention, “Family Health Conversations” (FamHC) was conducted in order to strengthen the health of families having relatives at residential home for older people.

**Objectives:**

The aims of this study were to evaluate the responses to the Family Health Conversations in families with a member living at a residential home for older people and to integrate the empirical results with a theoretical assumption upon which the intervention was based.

**Methods:**

A mixed methods research design was used. The Swedish Health-Related Quality of Life Survey and the Family Hardiness Index were administered before and 6 months after the intervention. Qualitative data was collected by semi-structured interviews with each family 6 months post-intervention. The sample included 10 families comprising 22 family members.

**Result:**

Main finding was that FamHCs helped family members process their feelings about having a member living at a residential home and made it easier for them to deal with their own situations. FamHCs helped to ease their consciences, improve their emotional well-being, and change their beliefs about their own insufficiency and guilt. Seeing problems from a different perspective facilitated the families’ thinking in a new way.

**Conclusion:**

These findings showed that FamHC could be an important type of intervention to improve family functioning and enhance the emotional well-being.

## INTRODUCTION

When a family member moves into or lives in a residential home for older people, it affects not only that resident, but also the entire family. Some studies have shown that relocation to a residential home is a distressing experience for family members [1-3]. This transition often occurs when the family’s burden of caring for their older relative increases beyond their capacity [4]. Family members have described feelings of relief after the initial move of the older person, but also feelings of loss, guilt, sadness [5, 6], and stress [7]. When the older person had moved to a residential home, relationships within the family can sometimes become troublesome, and complex new patterns of family relationships may arise [7]. Studies have shown that family members will remain involved in the lives of their sick relative following placement in residential homes for older people [8, 9], and as a result, their need for support is important [10].

Several studies suggest that families require encouraging and open communication and collaborative support from nurses during and after relocation to a residential home [11-14]. According to Bramble *et al*., [5], family members wish for a more communicative relationship with nurses based on non-judgmental collaboration, which can be achieved through Family Systems Nursing (FSN) interventions. FSN provides a model of care in which the resident and the resident’s family, rather than the resident alone, are recognized and formalized as the unit of care [15]. Viewing the family as a unit requires that attention be paid to the situation of the whole family, rather than only to the diseases and condition of the resident [16, 17] also when not all family members are present.

FSN is based on the assumption that health and illness affect all family members. The FSN goal is to maintain health and facilitate healing in the family in terms of functioning, beliefs, and meanings related to the problems described by the family [15]. In previous studies, families’ experiences after participating in FSN interventions showed that family interventions were appreciated by families [18-24]. To our knowledge, there have been no quantitative evaluations of effects or responses upon family members to an older person living in a residential home. Relocation to a residential home is for the old person and rest of the family described in studies as one of the most stressful events in life [6, 25].

The Family Health Conversation (FamHC), an FSN intervention, influenced by the Calgary Family Assessment Model (CFAM), the Calgary Family Intervention Model (CFIM) [15], and the Illness Beliefs Model (IBM) [26]. The FamHC is a systemic approach focused on the interactions and relationships between family members` beliefs and experiences in which each family member’s view is equally important [20, 27] and personal narratives and reflections are significant and emphasized. Narration is believed to have a profound effect on the healing process [28], and is closely joined together with reflections, which is thought to facilitate the emergence of new beliefs and the discovery of new alternatives or meanings that can also have an impact on the health [29].

Research into FamHc shows positive impact for families [19-22, 30-32]. Based on the increased evidence in the literature on positive outcomes of paying attention to families in health care, FamHC may facilitate building a trusting relationship with families that have a family member living in a residential home. This may result in families becoming more confident with the situation and lead to a better relationship with the nurses [33, 34]. A good relationship may also contribute to a psychosocial well-being of the family member living at the residential home [34, 35]. However, even if FamHCs is well grounded in theory and the empirical evidence of how it works in practice, more empirical studies are still needed before the intervention is implemented more broadly in different contexts. Using a mixed methods research approach when evaluating complex interventions, such as FamHC, enables an understanding as to whether or not an intervention works [36].

Given this background, we suggest *the following theoretical assumption* concerning responses to and effects of FamHCs: Family Health Conversations create a context for change and support the creation of new beliefs, new meanings, and new opportunities to manage the problems described by the family. This way of facilitating health will promote healing and support family changes and adaptations to restore balance and harmony in the family. Therefore, the aims of this study were to evaluate the responses to the Family Health Conversations (FamHCs) in families with a member living at a residential home for older people and to integrate the empirical results with a theoretical assumption upon which the intervention was based.

## MATERIALS AND METHODOLOGY

In this study, a mixed methods research design was used. Qualitative and quantitative data were integrated with an underlying theoretical assumption by using triangulation as a methodological metaphor [37, 38].

### Participants and Setting

The sample included families of residents in three residential homes for older people. The inclusion criterion was having a member living in one of the residential homes for older people where the intervention was conducted. The exclusion criterion was an inability to speak or read Swedish. Four families at each unit (12 families in total) were enrolled in the intervention. None of the residents participated in the FamHcs because of their condition or illness. Two families who participated in the FamHCs declined to participate in the follow-up interviews due to health-problems and lack of time, respectively. Consequently, 10 families comprising 22 family members were included in the study. The participating family members (20 women and 2 men) were 39–84 years old (Median = 55).

### Intervention

Prior to the intervention, three nurses from three residential homes for older people participated together in session related to FamHC. The education was a tailored and shortened version of a regular university course in FamHC [39]. The nurses learned about the theoretical basis of family-system nursing and practiced FamHC in role-playing exercises.

The theoretical assumptions of FamHC comprise a number of core components [20]. Reciprocity, humility, and respect for the families and their situation, their beliefs, and ideas are central to the intervention. A non-hierarchical approach between the nurse and the family is also essential in building a trusting relationship. A non-hierarchical approach is characterized by reciprocity, where the nurses and each family member are acknowledged as equally valid [27]. The intervention consisted of families participating in a series of three 1-hour conversations about every other week. Two nurses led each conversation, one nurse who had participated in the education and one nurse from the research group previously trained in FamHCs. The family members and the nurses created the conversational content together with the aim of finding alternative solutions to problems described by the families. During the first conversation, all family members were invited to tell their stories and encouraged to listen to each other’s. The second conversation was intended to focus on the problems and suffering identified in the first conversation. The third conversation focused on family strengths and resources for the future [27]. Two weeks after the last conversation, a closing letter was sent to all the family members. The letter provided the nurses’ reflections over the three conversations, acknowledging the families’ suffering and highlighting their resources [40].

### Qualitative Data Collection and Analysis

Semi-structured group interviews were conducted six months after the FamHCs intervention to collect qualitative data from each family [41]. Individual interviews were used in the two families that had only one participant. The interviews concerned families’ experiences of and reflections on participating in the FamHCs and followed an interview guide to ensure that questions relevant to the study aim were asked [42]. The family members were asked whether and how anything had changed in the family after their participation in the conversations and how the conversations had affected them. The interview started with an open question: “Can you tell me about your experiences of participating in the Family Health Conversations?” The interview continued with follow-up questions “Can you tell me whether or not the conversations have been helpful to you in your family, and if so, how?” “Can you tell me whether or not the conversations have contributed to a change in your family, and if so, how?” “Did you experience the conversation as positive, and if so, why / what / how?” “Did you experience the conversations as negative, and if so, why/what/how?” Probing questions, such as “Who?”, “When?” and “What do you mean?” were asked when necessary. The interviews, performed by one researchers who had not been involved in the intervention, took place in a conversation room in the particular residential home and lasted about 60 minutes. All interviews were tape-recorded and transcribed verbatim.

The interviews were analysed using qualitative content analysis [43] performed in several steps. First, all transcripts were read through several times in order to gain a sense of the whole [44]. The text was then reread and divided into meaning units, each representing a single unit of content [45]. The meaning units were then condensed and labelled with codes. Through comparing the codes for similarities and differences, two categories with seven related subcategories were abstracted. The analysis was an interprative ongoing process, in which the authors reflected until agreement was reached.

### Quantitative Data Collection and Analysis

The Family Hardiness Index (FHI) [46] was used to measure the intervention’s effect on family stress resistance and adaptation resources. The instrument consists of 20 statements scored on a 4-point Likert-type scale. In this study, the version consisting of the 3 subscales *Commitment* (the family´s ability to work together and the confidence in handling problems), *Challenge* (the family´s approach and attitude towards new experiences), and *Control* (the sense of being in control of family life) was used. A total score was calculated, ranging from 0 to 60, in which a higher score reflected higher family hardiness. The Swedish version of the FHI used in the study indicates good internal consistency (α = 0.86) for a newly published instrument [47].

The Swedish Health-Related Quality of Life Survey (SWED-QUAL) was used to measure the effect the intervention had on the families’ health-related quality of life. The instrument consists of 61 items calculated on 12 subscales reflecting different aspects of health-related quality of life. The response options are of the Likert type and offer four or five response alternatives. The scores are transformed into a 0–100 score index, with higher scores reflecting better functioning and well-being. The instrument shows good internal consistency (α = 0.78) for all subscales [48].

Comparisons of SWED-QUAL and FHI results from baseline to 6-month follow-up post intervention were made using Cohen’s *d* to calculate effect size. According to Cohen [49], benchmarks for effect size are 0.2, 0.5, and 0.8, respectively, for small, medium, and large effect sizes. A value > 0.25 was considered clinically significant [cf.50]. Clinical significance may be defined as a difference large enough to have an impact on a person's health [51]. Wilcoxon signed-rank were conducted to determine statistically significances between baseline and follow-up. A p-value <0.05 was considered statistically significant.

### Integration

To integrate the quantitative and qualitative results we used triangulation as a methodological metaphor as described by Erzberger and Kelle [37] and exemplified by Östlund et al. [38]. This method links theoretical assumptions to empirical findings and assigns the different data sources equal weights. The sides of the triangle represent relationships between the theoretical assumptions and the empirical findings originating from qualitative and quantitative data. Results that are found to be convergent, complementary, or divergent are reflected by different types of triangle [38].

### Ethics

Permission to conduct the study was given by the head of administration and the heads of the units. Written and verbal information concerning the study aims, voluntary participation, and confidentiality were given to the participants in accordance with research ethics. Consent to carry out and record the interview was sought from the families before they were included. The Regional Ethical Review Board approved the study (2011-335-31M).

## RESULTS

The results are reported in three sections: first, results of the qualitative interviews with family members; second, the results of the instruments SWED-QUAL and FHI; and third, the integration of the empirical results from interviews and instruments with the theoretical assumptions.

### Qualitative Findings 6 Months After the Intervention

The findings revealed two categories, *Improved family functioning* and *Enhanced emotional well-being*, reflecting the responses of the families after their participation in the FamHCs.

#### Improved Family Functioning

##### A deeper relationship within the family

Family members described an improved understanding within the family when they discovered what kinds of issues each family member was struggling with. After participation in the FamHCs, they felt they could share their grief and suffering, and this improved relationships within the family.

“We have always had a great relationship, but after the conversations we have come even closer to each other”.

##### Started dialoguing within the family

Family members described having more honest and deeper conversations after participating in the FamHCs. Having learned about the other family members’ views of the situation, they found it easier to talk to each other at home and to express what they were feeling without becoming too emotional.

“Now I understood how my mother looked at the problems compared to me.”

##### Shared responsibility within the family

Improvements in sharing responsibilities within the families was another result of participating in the FamHCs. Before the conversations the family members tried to arrange everything themselves. They described feeling that their burden of responsibility had lightened after sharing their experiences with each other, and they saw each other’s problems from a different perspective. Participants felt the family had become a team working together towards the same goals.

“We have become even stronger. We are a strong team when difficulties and such things arise.”

### Enhanced Emotional Well-Being

#### Feeling strengthened

The FamHCs gave the family members courage to express their needs and to ask for help not only outside the family but also within the family, and they felt this had improved their health. They described an improved life situation, a happier mood, and a brighter view of the situation than they had had before the conversations. Their previous attempt to cope with the new situation of having a sick family member living in a residential home had not gone well. The conversations had helped them to process their feelings about having a family member in a residential home.

“During the conversation I was allowed to speak freely, and it strengthened me.”

#### Gaining facilitating beliefs

After participating in the FamHC, the family members looked upon things in a different way and were more aware of their beliefs and emotions. The FamHCs made it possible for them to think differently, feel better about their situation, and move on with their lives.

“It’s easy to get stuck in a mind-set; I think the nurses helped us to think in a different way.”

#### Getting relief from sharing feelings

The family members expressed feeling relief after participating in the FamHCs and being able to share their feelings of grief and suffering with other members in the family: they were no longer alone in their sorrow. They also said that they had learned that expressing their feelings was a positive thing that helped to relieve their pressure, anxiety, and frustration and make the situation easier to deal with.

“The positive [thing] was to unburden one’s heart. It is packed [full] all the time, and it hurts so much.”

#### Easing a bad conscience

Participating in FamHCs helped the families to decrease the demands they put on themselves, such visiting the sick family member every day. Family members described living with a constant guilty conscience about their loved ones before the conversations. After participating in the FamHCs, they gained insight into the importance of allowing themselves to be happy. They felt that they had developed tools for how to think about and handle their feelings of insufficiency and guilt so they could ease their bad consciences.

“The most important [outcome] of these conversations is that I have been helped to manage my bad conscience.”

### Quantitative Results – Families’ Hardiness and Quality of Life Before and Six Months After the Intervention

The results of the families’ assessments showed tendencies of improvement concerning the subscale negative affect at follow-up. The result also showed a tendency that pain were scored lower and sleep problems were scored higher at follow-up compared to baseline, however, not to a significant extent. When calculating for effect size, the result showed a positive clinical significant effect in commitment, control, and total family hardiness at follow-up. Emotional well-being, negative affect, and sleep problems also proved to be showed a clinical significant effect, while pain showed a medium worsening effect at follow-up compared to baseline (Table **1**). These results indicate that the intervention improved the families control over their situation and their mental health but led to more pain among the participant.

### Integration of the Results on the Empirical Level and the Theoretical Assumptions

The qualitative and quantitative results were then integrated with the theoretical assumption. This integration, using the methodological metaphor of the triangle, of the empirical results and theoretical assumptions is presented in this section and illustrated in Fig. (**1**). In this study, we have interpreted the results from the quantitative and qualitative findings to be convergent, i.e. pointing to the same conclusion as the outcomes and responses demonstrating improvements in family functioning and health. When deductively testing the theoretical assumption and asking about families responses after participating in FamHC, the theoretical assumption were aligned with empirical results.

The theoretical assumption suggests that FamHCs will promote healing and sustain family health. This is supported by the quantitative findings. An improvement of hardiness was seen in the FHI total score and the subscale measuring commitment (families’ internal strengths and ability to work together) from baseline to 6-month follow-up. The result from SWED-QUAL showed increased emotional well-being in family members and decreased negative affect, negative affect include feelings of anxiety, sadness, nervousness, and tension, and these were decreased after participating in the FamHCs.

The qualitative results also support the theoretical assumption. The participants described improved cooperation, communication, and relationships within the family. They also said their families had become better able to share responsibilities. On a more personal level, participants described feeling stronger after the conversations and better able to process their feelings about having a family member living in a residential home. Anxiety and frustration were also relieved, which made it easier for them to deal with their situation. They felt their consciences were eased, and that led to their improved emotional well-being and created a change in their beliefs about their own insufficiency and guilt.

The part of the theoretical assumption that suggests that FamHC creates a context of change and supports the creation of new beliefs, new meaning, and new opportunities to manage problems was also supported by both the qualitative findings and the quantitative data. The families described how they had changed their ways of looking upon the situation and had begun to think in new ways. They had changed how they communicated with each other and had learned about how other family members viewed the situation. Furthermore, they saw problems from a different perspective, which made it possible to think differently about how to deal with their situation and continue with their lives. The quantitative data showed a clinical significant effect on the families’ abilities to take control of their family lives rather than being shaped by outside circumstances or events.

## DISCUSSION

In this study responses to the FamHCs in families with a member living at a residential home for older people were evaluated and integrated with a theoretical assumption of the intervention. The empirical findings, when integrated, supported the theoretical assumption. Consequently, there is no need to modify or expand our assumption underlying the FamHC intervention for older people and their families. The integration of the empirical findings showed that the intervention improved the families’ control over their situation, ability to work together, family function, and emotional well-being. These findings indicate that the FamHCs facilitates movement towards healing and sustained family health.

Having a family member move to a residential home for older people can be understood through the perspective of life transition as a passage from one life phase to another [52]. According to a previous study, family members’ experiences the relocation of an older family member to a residential home as a difficult and dynamic process [7]. The change in their everyday home lives when a family member moves to a residential home can be hard to cope with. According to the families’ statements, the family members’ earlier attempts to adjust to this situation had not been successful, as they had not been supported or prepared for the emotional turbulence of the transition. This is in line with other studies about families experiences of the transition to a residential home for older people [7, 53, 54]. Our results showed that after participating in the FamHCs, the families were better adapted to the new situation. The intervention started a process among the family members directed towards a transition gaining a new beginning and new perspective. The FamHCs facilitated the family members to reach a new point in life where they could feel more satisfied with life and accept their new situation. This transition process continues over time and can either lead to a movement towards health or render families more vulnerable [55]. Coping with transition is a dynamic process that includes different processes where health and perceived well-being are the outcomes [56]. Characteristics of periods of transition are instability, distress, and confusion. Early assessment and nursing interventions are very important to facilitating emotional well-being and healthy outcomes after a period of change in life pattern [55]. That is in line with our study, which showed increased family hardiness, emotional well-being, and a change in families’ way of thinking following the FamHC intervention. The findings from our study can be interpreted as increased interaction and connectedness after participating in the conversations, and family members described an enhanced understanding within the family. The conversations were found to have strengthened family cohesion; that is, after the intervention, participants were able to transfer their complex problems from an individual to a family level. They could share their grief and suffering with each other, this is in line with the study of Deist and Greeff [56].

Some patterns of response are known to facilitate the transition process. These patterns includes feeling connected (e.g., the need to feel connected to old friends and family and to health care professional) and interacting with others. According to Meleis et al. [57], properties that facilitate the transition process towards health and emotional well-being include community resources such as–support from partners and families and relevant information from health care providers. The FamHCs helped the family members manage their situation by accepting support from each other. The findings show an increase in the families’ sense of internal strengths and their ability to work together as a family.

Previous studies have demonstrated that it is a critical point in the transition process when individuals reach a state of stability in their new routines and lifestyles [57]. Our result showed that the family members’ previous attempts to adjust to their new situation had not been successful, but with the support of the FamHC, they learned to communicate more clearly within the family. Once the families in our study accepted that they were unable to change their circumstances, they were better able to cope with their situation. They made this adjustment in an attempt to manage the demands and challenges of their changed life situation. This led to an adaptation in their beliefs about how they could initiate changes in the family’ internal structures and deal with the new situation differently than before to restore balance and harmony in the family, this is also in line with the study of Greff, Vansteenwegen, and Ide [58].

Our findings can be interpreted to mean that the FamHCs made the family members’ various beliefs visible and thus started a process that may have facilitated changes in their ways of thinking. Family members felt that after participating in the FamHCs their bad conscience towards their older family member had been eased. By participating in the FamHCs, they seem to have been helped in their transition and gave themselves permission to come to terms with the new situation of their family member living in a residential home. Our findings can also be understood in the light of adjustment and adaptation as described in the resiliency model of family stress. A family’s state of adjustment is influenced by the family’s assessment of the stressor, its vulnerability, and its overall family functioning, problem solving, coping strategies, and available resources. When the problems and challenges are too difficult to manage, the family may become maladjusted and can face a crisis. When the balance in family functions, interpersonal relations, and individual well-being are favoured, adaptation is achieved [59].

The families’ capability for resilience in a changed life situation in this study was favoured by positive beliefs, family roles, family function, and communicative problem-solving abilities [60]. Similar to Walsh [61], we interpreted the results and identified three components that support our theoretical assumption and form family resilience: belief systems, organizational patterns, and communication processes. Belief systems in the family have a strong impact on family functioning and are an important contributor to resilience. Family patterns such as family rules, family functioning, and family cohesion all influence how family members respond to changes. The third domain about the communication process favours open communication, supported by a climate of empathy, trust, and tolerance of all family members’ views about the situation, which enables family members to share feelings with each other. The findings in our study indicate that the families strengthen this ability by participating in the FamHCs. Families’ beliefs were shared and challenged within the conversations, which made them each aware of their own beliefs as well as those of the other members. The FamHCs helped the families integrate the changes imposed by the major life event of having an older family member live in a residential home and communicate in a more honest and deeper way. To accept and manage this situation, it was important for the family to adapt to the situation [58]. Our results indicate that the FamHC intervention improved the families’ control over their situation and their family’s health.

### Methodological Considerations

In this study, we used mixed methods research design, based on integrating qualitative and quantitative data with the intervention’s theoretical assumptions. The methodological metaphor of triangulation helped to describe the logical relations between the qualitative and quantitative findings on the empirical level and the explicitly stated assumptions on the theoretical level. A mixed methods research is suitable to investigate effects in complex interventions since findings from one method strengthen from the other method and a combination of the two methods help explain the findings and neither method alone will answer the study object [62].

The sample size was 22 family members, which can be assumed as a sufficient number for the qualitative analysis since the interviews were rich in content and gave a good variation in their responses. However, this is a small sample when considering the quantitative analysis and we therefore used a non-parametric test since there can be questions concerning the normality of the data. As statistical significance is just a statement about likelihood of findings being due to chance and the *p* value on its own provides no information about the meaning of the results to clinical practice or the overall importance [63], in the study design, a measure to establish clinical significance were chosen. Thus, Cohen’s *d* >0.25 was considered as a benchmark for the intervention is clinically significant, as proposed by Wolf [50]. We consistently used validated instruments in the quantitative part of the study. To strengthen the trustworthiness of the qualitative data, the person performing the interviews with the families had not participated in the conversations with the families. To strengthen the credibility of the qualitative data all authors discussed each step together in the analysis process until a consensus was reached. All authors reflected independently upon the findings to ensure that nothing were overlooked.

When including families none of the family members living at the residential homes were included due to illness, fatigue, frailty and communication difficulties caused by dementia, which could have caused confusion to the residents if participated. Moreover, there was a predominance of female family members which may have affected the results. Even though the sample size was small, the choice of using mix-methods design integrating qualitative and quantitative findings was seen as a strength in this study. We achieved a more comprehensive in-depth information about the responses to and effects of the FamHC on the family members. Even so, although we believe that our results are transferable, we recommend that generalizations to other populations should be made cautiously.

### Conclusion and Implications for Practice

From the integration of the empirical results and our theoretical assumptions it can be concluded that participating in FamHCs facilitates a context for change and supports the creation of new beliefs, new meanings, and new opportunities in relation to the problems described by families with a member living in a residential home for older people. The family members’ abilities to manage the transition of moving their sick member from home to assisted living improved with the intervention. This study shows the importance of nurses caring not only for the resident but also for the whole family. Therefore, FamHCs should be offered as a part of standard care to support family health. For nurses it is essential to expand the approach from patient-centred care to an approach in which the family is viewed as a unit of care, as in FSN. Family Systems Nursing interventions such as FamHC should be considered as a natural responsibility for nurses to support family well-being. Consequently, opportunities for education in training for and conducting this kind of family intervention should be considered, as there is a further need for knowledge transfer in this area from theory to practice. Even if this study adds to the evidence base of FSN interventions in general and FamHCs in particular more empirical research is still needed to strengthen the research evidence in this specific context.

## Figures and Tables

**Fig. (1):**
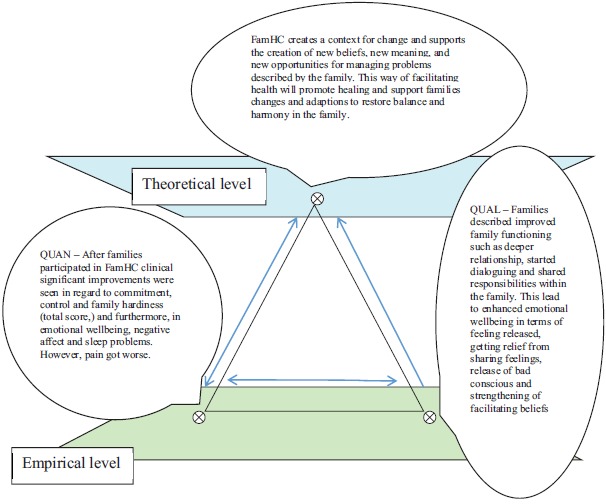
Triangulation diagram of the logical relationships between the theoretical assumption, the quantitative data from SWED-QUAL and FHI, and qualitative findings from the interviews.

**Table 1:** Measures before and after intervention (n = 22).

		Baseline Mean (SD)			Follow-up Mean (SD)	Cohen’s *d*		*P*	
FHI									
Total (range 0-60)			43.14 (6.92)	45.64 (7.09)		0.27			0.210
Commitment (range 0-24)			16.82 (4.96)	18.41 (4.57)		0.26			0.222
Challenge (range 0-18)			11.91 (2.84)	12.09 (1.90)		0.06			0.777
Control (range 0-18)			14.41 (2.26)	15.14 (1.86)		0.39			0.066
SWED-QUAL (range 0-100)									
Physical functioning	82.98 (18.69)			80.34 (20.39)		0.04			0.850
General health	74.12 (20.71)			77.44 (17.80)		0.13			0.573
